# Strengthening STD Screening Programs: Comprehensive Evaluation of High-Throughput Immunoassays for HIV and Syphilis Detection

**DOI:** 10.3390/microorganisms14061302

**Published:** 2026-06-09

**Authors:** Ahmed Ismail, Shaden Abunasser, Israa M. Salameh, Mazen Najib Abouassali, Manal Elshaikh, Ibrahim Wissam Karimeh, Mohammed Abdelfatah Ibrahim, Mutaz Mohamed Ali, Ibrahim Al Shaar, Parveen Banu Nizamuddin, Salma Younes, Hadi M. Yassine, Laith J. Abu-Raddad, Nadin Younes, Gheyath K. Nasrallah

**Affiliations:** 1 Laboratory Section, Medical Commission Department, Ministry of Public Health, Doha 42, Qatar; aismail@moph.gov.qa (A.I.); mnajib@moph.gov.qa (M.N.A.); mibrahim1@moph.gov.qa (M.E.); ikarime@moph.gov.qa (I.W.K.); mabdulfatah@moph.gov.qa (M.A.I.); mmohamoud@moph.gov.qa (M.M.A.); ialshaar@moph.gov.qa (I.A.S.); 2 Department of Biomedical Science, College of Health Sciences, QU Health, Qatar University, Doha 2713, Qatar; sa1708343@student.qu.edu.qa (S.A.); sy1203986@student.qu.edu.qa (S.Y.); hyassine@qu.edu.qa (H.M.Y.); 3 Biomedical Research Center, Qatar University, Doha 2713, Qatar; israa.salameh46@gmail.com (I.M.S.); parveen.n@qu.edu.qa (P.B.N.); 4 Infectious Disease Epidemiology Group, Weill Cornell Medicine-Qatar, Cornell University, Qatar Foundation—Education City, Doha 24144, Qatar; lja2002@qatar-med.cornell.edu; 5 World Health Organization Collaborating Centre for Disease Epidemiology Analytics on HIV/AIDS, Sexually Transmitted Infections, and Viral Hepatitis, Weill Cornell Medicine-Qatar, Cornell University, Qatar Foundation-Education City, Doha 24144, Qatar; 6 Department of Healthcare Policy and Research, Weill Cornell Medicine, Cornell University, New York, NY 10065, USA

**Keywords:** HIV, syphilis, chemiluminescent immunoassay, screening, diagnostics, public health, MAGLUMI, ARCHITECT, VITROS, INNO-LIA, RPR, PCR

## Abstract

Fourth-generation immunoassays are widely used for HIV and syphilis screening; however, false-reactive results may increase confirmatory testing and operational burden in high-throughput laboratories. This study evaluated the comparative performance of automated chemiluminescent immunoassays (MAGLUMI^®^ HIV Ab/Ag Combi (Snibe Diagnostics Co. Ltd., Shenzhen, China), VITROS^®^ ECiQ HIV Combo (Ortho Clinical Diagnostics, Raritan, NJ, USA), MAGLUMI^®^ Syphilis (Snibe Diagnostics Co. Ltd., Shenzhen, China), and ARCHITECT^®^ Syphilis TP (Abbott Diagnostics, Abbott Park, IL, USA) within a routine diagnostic algorithm, incorporating antibody differentiation immunoassays (INNO-LIA^®^ HIV I/II Score (Fujirebio Europe N.V., Ghent, Belgium) and HIV-1 RNA PCR where applicable. A total of 240 archived serum samples for HIV testing and 180 for syphilis testing were analyzed. Agreement-based performance measures including sensitivity, specificity, overall percent agreement (OPA), and Cohen’s kappa (κ) were calculated as comparator-based estimates reflecting concordance within the routine diagnostic algorithm rather than absolute diagnostic accuracy against a universal reference standard. For comparisons with HIV-1 RNA PCR, positive and negative concordance rates are reported to reflect agreement between assays detecting different biological targets. Among samples with definitive (positive or negative) results, the MAGLUMI^®^ HIV Ab/Ag Combi assay showed complete agreement with INNO-LIA^®^ HIV I/II Score (κ = 1.00) and high agreement with PCR within the ARCHITECT^®^ HIV Ag/Ab Combo-reactive subset (κ = 0.90). The VITROS^®^ ECiQ HIV Combo assay demonstrated high agreement with INNO-LIA^®^ HIV I/II Score (κ = 0.916) and substantial agreement with PCR (κ = 0.715), with a lower negative concordance rate with PCR observed in the ARCHITECT-reactive subset. A parallel five-modality analysis of 11 discordant samples applying the CDC 2014 algorithm demonstrated that all three immunoassay platforms successfully detected confirmed HIV-seropositive individuals with controlled viremia despite negative PCR, while MAGLUMI^®^ HIV Ab/Ag Combi produced fewer false-reactive results than both ARCHITECT^®^ and VITROS^®^ in this discordant subset. Additionally, two cases showed INNO-LIA^®^ indeterminate results with positive PCR, consistent with acute HIV infection during the early seroconversion stage; all three immunoassay platforms produced signals above the non-reactive threshold in both cases. For syphilis testing, both MAGLUMI^®^ Syphilis and ARCHITECT^®^ Syphilis TP assays showed complete agreement with INNO-LIA^®^ Syphilis Score among samples with definitive results (κ = 1.00). In contrast, the RPR assay showed reduced positive predictive value (49.4%) and moderate agreement with INNO-LIA^®^ Syphilis Score (κ = 0.52). Automated chemiluminescent immunoassay (CLIA) platforms demonstrated high agreement within a structured diagnostic algorithm in a high-throughput screening setting. Differences in assay performance were observed across platforms, particularly with respect to discordant results in the ARCHITECT-reactive PCR-evaluated subset for HIV and non-treponemal concordance for syphilis. These platforms may support more efficient laboratory workflows; however, findings should be interpreted within the context of comparator-based classification rather than absolute diagnostic accuracy.

## 1. Introduction

Sexually transmitted diseases (STDs) remain a major global health challenge, contributing substantially to morbidity, mortality, and socioeconomic burden [1]. Among these infections, human immunodeficiency virus (HIV), the causative agent of acquired immunodeficiency syndrome (AIDS), and *Treponema pallidum* (TP), the bacterium responsible for syphilis, are of particular concern. In 2024, an estimated 1.3 million new HIV infections occurred worldwide, adding to the 40 million individuals living with HIV [2]. Similarly, syphilis continues to affect global populations, with more than 12 million new cases annually, the majority in low- and middle-income countries [3]. Both infections are frequently asymptomatic in early stages, facilitating transmission and delaying diagnosis. If untreated, HIV leads to progressive immunodeficiency, while syphilis may progress through primary, secondary, latent, and tertiary stages, potentially resulting in severe neurological and cardiovascular complications [4,5,6].

Co-infection with HIV and syphilis is common and clinically significant, as syphilis increases susceptibility to HIV acquisition and may accelerate disease progression [7]. Studies suggest that up to 10% of individuals with syphilis acquire HIV within five years [8]. Accordingly, international guidelines recommend routine HIV screening among patients diagnosed with syphilis and repeat testing in high-risk populations [9]. Substantial epidemiological and mechanistic evidence supports the role of syphilis in enhancing HIV transmission [10].

**Figure 1:**
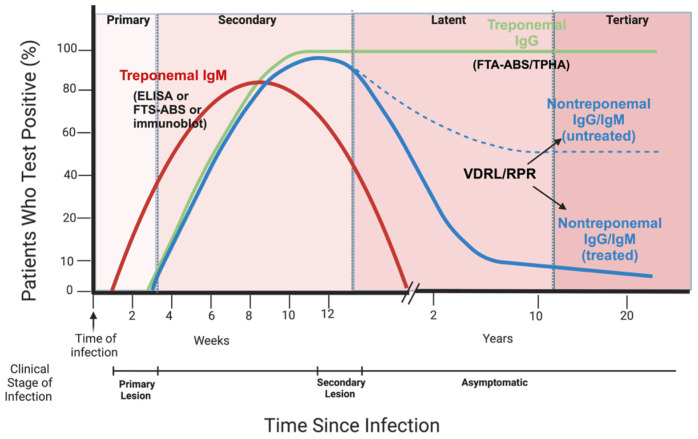
Progression of syphilis serology across the stages of infection (adapted from [31]). The figure illustrates the serological response to *Treponema pallidum*, comparing treponemal and non-treponemal test reactivity throughout disease progression. Treponemal tests detect IgG/IgM antibodies against T. pallidum antigens, typically becoming positive 2–4 weeks after exposure and remaining reactive for life, indicating exposure rather than active infection. Non-treponemal tests measure antibodies directed against lipoidal antigens released from damaged host cells, reflecting disease activity and treatment response as titers decline following therapy. Created in BioRender. Younes, N. (2026) https://BioRender.com/i01w2j5.

**Figure 2:**
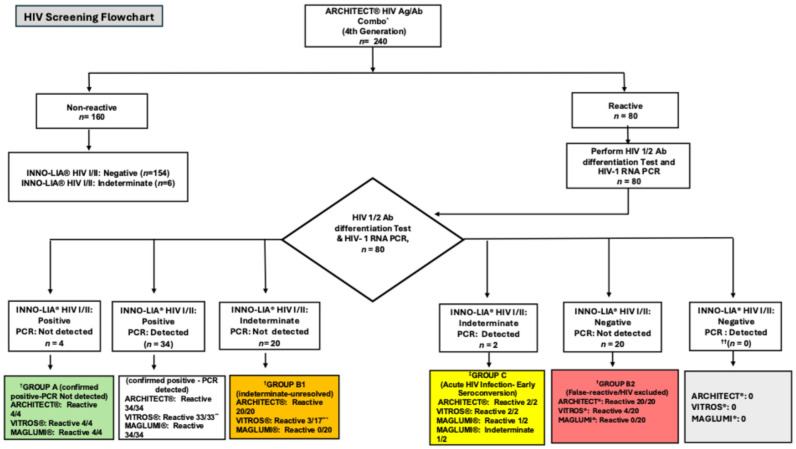
HIV screening and diagnostic algorithm at the Medical Commission (Qatar) with study sample flow overlay (*n* = 240; 2022–2023). Screening was performed using the fourth-generation ARCHITECT^®^ HIV Ag/Ab Combo immunoassay. Reactive specimens (*n* = 80) underwent INNO-LIA^®^ HIV I/II Score antibody differentiation and HIV-1 RNA PCR; non-reactive specimens (*n* = 160) were additionally tested by INNO-LIA^®^ HIV I/II Score. Final interpretation applied the CDC 2014 HIV Diagnostic Algorithm. Group A (*n* = 4): confirmed HIV-seropositive, PCR negative (controlled viremia). Group B1 (*n* = 20): INNO-LIA^®^ indeterminate, unresolved. Group B2 (*n* = 20): HIV infection excluded. Group C (*n* = 2): acute HIV infection, early seroconversion stage. * Ag: Antigen and Ab: Antibody; ** One sample excluded-insufficient volume (VITROS^®^); *** Three samples excluded-insufficient volume (VITROS^®^); ^†^ 11 discordant samples underwent parallel five-modality analysis, ARCHITECT^®^, MAGLUMI^®^, VITROS^®^, INNO-LIA^®^ and PCR, Group A (*n* = 4), B1 (*n* = 3) and B2 (*n* = 4) (see Section 3.5 and Table S3); ^††^ No cases of INNO-LIA^®^ negative with PCR-detected HIV-1 RNA were observed among the 80 ARCHITECT^®^-reactive samples, confirming that all truly infected individuals retained at least partial serological reactivity detectable by antibody differentiation testing. ^‡^ Group C (*n* = 2): INNO-LIA^®^ indeterminate with PCR-detected HIV-1 RNA, consistent with acute HIV infection during the early seroconversion stage.

Diagnosis of HIV relies on a range of assays, including rapid diagnostic tests (RDTs), Enzyme-Linked Immunosorbent Assays (ELISAs), chemiluminescent immunoassay (CLIAs) and Nucleic Acid Tests (NATs). In contrast, laboratory diagnosis of syphilis primarily depends on serology, as direct detection methods are rarely used in routine settings [11]. Serological assays are classified into non-treponemal tests (e.g., RPR, VDRL), which reflect disease activity, and treponemal tests (e.g., TPPA, TPHA, FTA-ABS, ELISA, CLIA), which typically remain positive for life and cannot distinguish active from past infection [6,11,12]. As illustrated in Figure 1, combining both test types improves diagnostic accuracy across disease stages. In contemporary HIV diagnostic practice, infection status is determined through a structured diagnostic algorithm, as illustrated in Figure 2, rather than reliance on a single reference assay. Current guidelines recommend fourth-generation antigen/antibody immunoassays for initial screening followed by HIV-1/HIV-2 antibody differentiation immunoassays for reactive specimens. According to the HIV diagnostic algorithm introduced by the U.S. Centers for Disease Control and Prevention (CDC) in 2014, nucleic acid testing (NAT) is primarily used to resolve discordant or indeterminate serologic results and to detect acute HIV infection rather than being applied universally to all screened specimens. In this algorithmic framework, antibody differentiation immunoassays serve as the confirmatory step for reactive screening results [13,14]. Similarly, syphilis testing strategies rely on algorithm-based approaches that combine treponemal and non-treponemal assays to support accurate diagnosis across different disease stages.

In Qatar, the Medical Commission (MC), operating under the Ministry of Public Health (MoPH), screens all newcomers to prevent the introduction and spread of infectious diseases including HIV and syphilis [15]. For HIV screening, the MC utilizes the fourth-generation ARCHITECT^®^ HIV Ag/Ab Combo assay (Abbott Diagnostics, Abbott Park, IL, USA) as the primary screening test. For reactive cases, a newly collected specimen is obtained for supplemental testing using INNO-LIA^®^ HIV I/II Score antibody differentiation assay and for HIV-1 RNA PCR in accordance with the diagnostic algorithm.

While this approach is robust, it is associated with a relatively high false-reactive rate of ARCHITECT^®^ HIV Ag/Ab Combo, necessitating repeated testing, increasing costs, and placing psychological and logistical burdens on patients and the healthcare system [16]. Reduced specificity may arise from biological and analytical factors, including heterophile antibodies, autoimmune conditions, polyclonal B-cell activation, and other cross-reactive serum components that produce weak reactive signals [17]. Participation in HIV vaccine trials may also lead to vaccine-induced seropositivity (VISP), and rare transient cross-reactivity following certain viral vaccinations, including COVID-19 vaccines, has been reported, although such findings are uncommon and assay-dependent [18,19,20]. Additionally, chemiluminescent signal amplification may accentuate minimal non-specific binding. In low-prevalence screening populations, even small decreases in specificity can markedly reduce positive predictive value, increasing the proportion of screening-reactive results not confirmed by supplemental testing [21]. On the other hand, antibody differentiation assays such as INNO-LIA^®^ HIV I/II Score may yield indeterminate results, which can delay definitive diagnosis and increase the need for repeat testing [16,22,23].

For syphilis, the MC follows a traditional algorithm, using the non-treponemal Rapid Plasma Reagin (RPR) test for screening and confirming with a treponemal-specific assay, typically the ARCHITECT^®^ Syphilis TP assay. However, non-treponemal assays detect anti-cardiolipin antibodies rather than pathogen-specific antibodies and are therefore susceptible to biological false reactivity. Conditions such as autoimmune disorders, pregnancy, and certain infectious diseases may produce false-reactive results [24], while treponemal tests remain positive after prior infection, limiting their ability to distinguish active from past disease [25].

Newer fully automated chemiluminescent immunoassays (CLIAs) may help address these limitations by improving assay consistency and workflow efficiency. The fourth-generation MAGLUMI^®^ HIV Ab/Ag Combi and MAGLUMI^®^ Syphilis assays, along with the VITROS^®^ ECiQ HIV Combo assay, which employs enhanced chemiluminescence to detect HIV-1/2 antibodies and p24 antigen, have demonstrated high sensitivity and specificity in hospital- and clinical laboratory-based evaluations involving routine diagnostic and screening specimens [26,27,28]; however, their performance in high-throughput, population-level screening program in Qatar has not been previously evaluated.

Therefore, this study aimed to evaluate the comparative performance of MAGLUMI^®^ HIV Ab/Ag Combi, MAGLUMI^®^ Syphilis, and VITROS^®^ ECiQ HIV Combo against the widely used ARCHITECT^®^ HIV Ag/Ab Combo and ARCHITECT^®^ Syphilis TP assays and established supplemental methods including INNO-LIA^®^ HIV I/II Score, INNO-LIA^®^ Syphilis Score, and HIV-1 RNA PCR within the diagnostic workflow used in the Medical Commission screening program [29,30]. By analyzing samples from a large and diverse screened population in Qatar, this study provides insights into assay agreement and operational performance in a real-world, high-volume screening setting.

## 2. Materials and Methods

### 2.1. Study Population, Study Design, and Ethical Approval

Serum samples were obtained from the Medical Commission (MC) laboratory, where they had been collected, tested, and archived as part of the routine visa entry infectious disease screening program for expatriates in Qatar. This retrospective study used only archived specimens and involved no direct or indirect interaction with human participants. Accordingly, the requirement for informed consent was waived by the Qatar University Institutional Review Board (QU-IRB), and the study was reviewed and approved under protocol number QU-IRB 017/2024-E.

**Figure 3:**
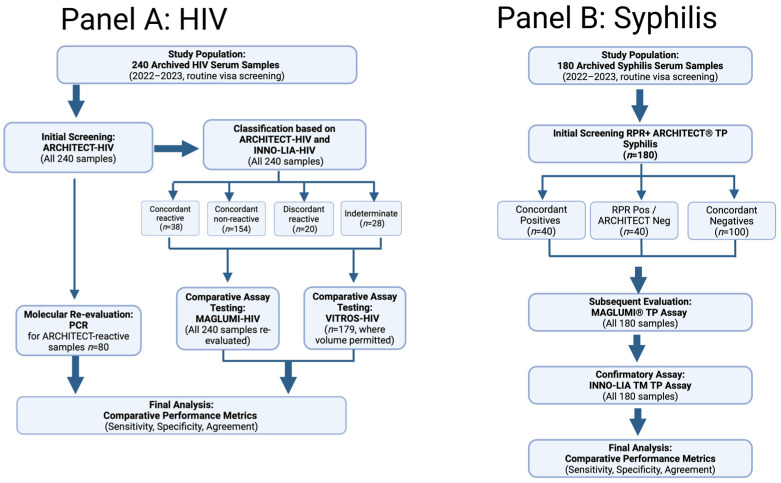
Schematic representation of the retrospective study design and diagnostic comparison workflow. Archived serum samples from the Medical Commission screening program were classified according to routine diagnostic algorithms and re-evaluated using automated chemiluminescent immunoassays. Panel A (HIV) illustrates the comparison of the MAGLUMI^®^ HIV Ab/Ag Combi and VITROS^®^ ECiQ HIV Combo assays with the INNO-LIA^®^ HIV I/II Score antibody differentiation assay and HIV-1 RNA PCR within the diagnostic framework. Panel B (Syphilis) illustrates the comparison of the MAGLUMI^®^ Syphilis and ARCHITECT^®^ Syphilis TP assays with INNO-LIA^®^ Syphilis Score as a treponemal comparator, with RPR serving as a non-treponemal comparator. Agreement-based performance measures, including sensitivity, specificity, overall percent agreement, and Cohen’s kappa, were calculated. Sensitivity and specificity were estimated relative to classification within the routine diagnostic algorithm using established comparator assays. Classification reflects concordance and discordance between assays and does not represent a definitive reference standard. Created in BioRender. Younes, N. (2026) https://BioRender.com/wordl8i.

A schematic overview of the study design is presented in Figure 3, which consists of two panels: Panel A (HIV workflow) and Panel B (Syphilis workflow). Panel A illustrates the study design used for the comparative evaluation of the MAGLUMI^®^ HIV Ab/Ag Combi and VITROS^®^ ECiQ HIV Combo assays within the Medical Commission (MC) laboratory. The study population consisted of 240 archived serum samples collected during routine MC screening between 2022 and 2023. Samples were selected from the Medical Commission archive to include a representative spectrum of concordant, discordant, and indeterminate result profiles across the testing modalities evaluated, in order to comprehensively assess assay performance across the full range of diagnostic outcomes encountered in routine practice. This intentionally enriched selection design was chosen to maximize the informative value of the comparison; however, it does not reflect the prevalence distribution of an unselected routine screening population. Accordingly, positive predictive values and specificity estimates should be interpreted in the context of this study design rather than as population-level diagnostic performance measures, consistent with the algorithm-based framework applied throughout this study. All samples were initially screened using the ARCHITECT^®^ HIV Ag/Ab Combo assay and subsequently categorized according to the combined results of ARCHITECT^®^ HIV Ag/Ab Combo and the INNO-LIA^®^ HIV I/II Score antibody differentiation assay. ARCHITECT-reactive specimens underwent reflex HIV-1 RNA PCR testing as part of the diagnostic workflow. For comparative evaluation, all samples were re-tested using the MAGLUMI^®^ HIV Ab/Ag Combi assay, while the VITROS^®^ ECiQ HIV Combo assay was performed on available specimens where sufficient sample volume was present. Panel B depicts the study design applied for the evaluation of the MAGLUMI^®^ Syphilis assay. A total of 180 archived serum samples, including both concordant and discordant results between the Rapid Plasma Reagin (RPR) and ARCHITECT^®^ Syphilis TP assays, were selected from routine screening specimens. All samples were tested using the MAGLUMI^®^ Syphilis assay, and results were compared with those obtained using the INNO-LIA^®^ Syphilis Score assay as a treponemal comparator.

To ensure participant confidentiality, all samples were anonymized prior to analysis and assigned unique study codes. For HIV analyses, specimens were categorized according to the results obtained with the ARCHITECT^®^ HIV Ag/Ab Combo screening assay and the INNO-LIA^®^ HIV I/II Score antibody differentiation assay. Samples reactive by both assays were coded as PP, samples reactive by ARCHITECT^®^ but negative by INNO-LIA as PN, samples reactive by ARCHITECT^®^ with indeterminate INNO-LIA as PI, samples non-reactive by ARCHITECT^®^ with indeterminate INNO-LIA results as NI, and samples negative by both assays as N. The same coding approach was applied to syphilis samples based on RPR and ARCHITECT^®^ Syphilis TP results. These classifications were used to structure the analytical dataset and facilitate comparative evaluation.

### 2.2. HIV Testing Workflow

In large-scale screening programs, HIV infection status is determined through sequential diagnostic algorithms rather than through a single universal reference test. Accordingly, the MC laboratory follows a diagnostic framework aligned with the 2014 CDC HIV diagnostic testing algorithm, incorporating fourth-generation antigen/antibody screening, antibody differentiation testing, and reflex nucleic acid amplification testing [13,14].

Within this framework, the INNO-LIA^®^ HIV I/II Score assay serves as the antibody differentiation step, while HIV-1 RNA PCR is used to resolve discordant or indeterminate results among ARCHITECT^®^ HIV Ag/Ab Combo-reactive specimens. Because HIV-1 RNA PCR testing is performed only for ARCHITECT-reactive specimens according to the MC diagnostic protocol, assay performance in this study was evaluated within an algorithm-based framework rather than against a universal reference standard.

Retrospective data were collected from individuals screened for HIV at the MC laboratory between 2022 and 2023 using the ARCHITECT^®^ HIV Ag/Ab Combo assay. A total of 240 archived serum samples demonstrating concordant, discordant, and indeterminate results between ARCHITECT^®^ HIV Ag/Ab Combo and INNO-LIA^®^ HIV I/II Score were selected for analysis. These included samples reactive by both assays (*n* = 38, samples), non-reactive by both assays (*n* = 154), reactive by ARCHITECT^®^ HIV Ag/Ab Combo but negative by INNO-LIA^®^ HIV I/II Score (*n* = 20), reactive by ARCHITECT^®^ HIV Ag/Ab Combo with indeterminate INNO-LIA^®^ HIV I/II Score results (*n* = 22), and samples non-reactive by ARCHITECT^®^ HIV Ag/Ab Combo with indeterminate INNO-LIA^®^ HIV I/II Score results (*n* = 6) (Figure 3).

All selected samples were re-tested using the MAGLUMI^®^ HIV Ab/Ag Combi. In addition, all ARCHITECT^®^ HIV reactive samples (*n* = 80) underwent HIV-1 RNA PCR testing as part of the diagnostic workflow. PCR served as a supplementary molecular comparator within this ARCHITECT-reactive subset, rather than as a universal reference standard. The VITROS^®^ ECiQ HIV Combo assay was also performed where sufficient specimen volume was available. Due to sample volume limitations, VITROS testing was completed for 179 of the 240 samples, including 76 of the 80 ARCHITECT-reactive specimens; four ARCHITECT-reactive samples were excluded from VITROS^®^ testing due to insufficient volume.

### 2.3. Comparator Selection and Reference Framework

The selection of comparator assays was based on the routine diagnostic algorithm implemented at the Medical Commission rather than on analytical performance alone. International guidelines from the Centers for Disease Control and Prevention (CDC) recommend a multi-step diagnostic strategy comprising an initial fourth-generation screening assay followed by an antibody differentiation immunoassay, with nucleic acid amplification testing (NAT/PCR) reserved for discordant or indeterminate results [13,14].

In this context, the INNO-LIA^®^ HIVI/II Score was used as the antibody differentiation comparator in accordance with the Medical Commission laboratory testing protocol. Although nucleic acid amplification testing (NAT/PCR) provides high analytical sensitivity, its use as a universal reference standard in large-scale screening programs is limited by cost, turnaround time, and operational constraints. Therefore, PCR was incorporated as a supplementary molecular comparator rather than a universal reference method, ensuring that assay comparisons reflect real-world implementation within the MC screening workflow.

### 2.4. HIV Diagnostic Assays

#### 2.4.1. ARCHITECT^®^ HIV Ag/Ab Combo Assay

The ARCHITECT^®^ HIV Ag/Ab Combo assay (Abbott Diagnostics, Abbott Park, IL, USA) is a fourth-generation automated immunoassay that detects HIV-1 p24 antigen and antibodies to HIV-1/2 using chemiluminescent microparticle immunoassay (CMIA). Results are expressed as signal-to-cutoff (S/CO) values, with ≥1.00 considered reactive according to the manufacturer’s instructions [32].

#### 2.4.2. MAGLUMI^®^ HIV Ab/Ag Combi CLIA Assay

The MAGLUMI^®^ HIV assay (Snibe Diagnostics Co. Ltd., Shenzhen, China) is a two-step sandwich chemiluminescent immunoassay (CLIA) for qualitative detection of HIV-1 p24 antigen and antibodies to HIV-1/2 in serum or plasma. Serum samples are incubated with magnetic microbeads coated with recombinant HIV antigens and anti-p24 monoclonal antibodies. Following washing, ABEI-labeled conjugates form immune complexes, and chemiluminescence is measured in relative light units (RLUs). Results are automatically interpreted by the analyzer [33].

#### 2.4.3. VITROS^®^ ECiQ HIV Combo Assay

The VITROS^®^ ECiQ HIV Combo assay (Ortho Clinical Diagnostics, Raritan, NJ, USA) [34] is a fully automated fourth-generation CLIA that detects HIV-1 (groups M and O), HIV-2 antibodies, and HIV-1 p24 antigen. The assay uses a two-stage immunometric technique in which HIV antibodies or antigen in the sample bind to biotinylated HIV recombinant antigens or antibodies immobilized on streptavidin-coated wells. Detection is based on enhanced chemiluminescence, with results expressed as signal-to-cutoff (S/CO) ratios (≥1.00 reactive). Reactive samples are retested in duplicate, and persistent reactivity is further evaluated using INNO-LIA^®^ HIV I/II Score antibody differentiation assay and the COBAS^®^ AmpliPrep/COBAS^®^ TaqMan^®^ HIV-1 Test (Roche Molecular Systems, Inc., Branchburg, NJ, USA), version 2.0, in accordance with the MC algorithm.

#### 2.4.4. INNO-LIA^®^ HIV I/II Score Confirmatory Assay

The INNO-LIA^®^ HIV I/II Score (Fujirebio Europe N.V., Belgium) is a line immunoassay (LIA) using recombinant and synthetic HIV-1/2 peptides. After incubation and washing, bound antibodies are detected using alkaline-phosphatase-conjugated anti-human IgG. Results are interpreted using LiRAS for Infectious Diseases v4.00 CE [35].

#### 2.4.5. COBAS^®^ AmpliPrep/COBAS^®^ TaqMan^®^ HIV-1 Test, Version 2.0

The COBAS^®^ AmpliPrep/COBAS^®^ TaqMan^®^ HIV-1 Test (Roche Molecular Systems, Inc., Branchburg, NJ, USA) is a nucleic acid amplification assay that quantifies HIV-1 RNA in plasma by targeting the gag and LTR regions. The quantification range is 20–10,000,000 copies/mL [36].

### 2.5. Syphilis Testing Workflow

A separate set of 180 archived serum samples was selected from routine Medical Commission (MC) screening specimens, representing concordant and discordant results between RPR and ARCHITECT^®^ Syphilis TP assays. This included concordant reactive (*n* = 40), discordant samples (RPR-reactive/ARCHITECT non-reactive; *n* = 40), and concordant non-reactive samples (*n* = 100). All samples were retested using the MAGLUMI^®^ Syphilis assay. The results were compared with those obtained using the INNO-LIA^®^ Syphilis Score assay as a treponemal comparator, with RPR serving as a non-treponemal comparator within the diagnostic algorithm (Figure 3). Performance was evaluated based on classification within the routine diagnostic algorithm using established comparator assays.

### 2.6. Syphilis Diagnostic Assays

#### 2.6.1. ARCHITECT^®^ Syphilis TP Assay

The ARCHITECT^®^ Syphilis TP assay (Abbott Diagnostics, USA) uses chemiluminescent microparticle immunoassay (CMIA) to detect *Treponema pallidum* antibodies. Serum or plasma samples are incubated with TP-coated microparticles, followed by acridinium-labeled anti-human IgG/IgM conjugates. The results are expressed as S/CO ratios, with ≥1.00 interpreted as reactive. The assay was performed following the manufacturer’s protocol [37].

#### 2.6.2. MAGLUMI^®^ Syphilis Assay

The MAGLUMI^®^ Syphilis assay (Snibe Diagnostics Co. Ltd., Shenzhen, China) is a sandwich CLIA for detecting total antibodies against *Treponema pallidum* in serum or plasma. Serum is incubated with magnetic microbeads coated with recombinant *T. pallidum*-specific recombinant antigens and ABEI-labeled conjugates. Chemiluminescence is measured in RLUs, and results ≥1.0 mIU/mL are considered positive.

#### 2.6.3. INNO-LIA^®^ Syphilis Score Assay

The INNO-LIA^®^ Syphilis Score (Fujirebio Europe N.V., Ghent, Belgium), is a line immunoassay (LIA) used for the simultaneous detection of anti-treponemal antibodies. The assay incorporates three recombinant *Treponema pallidum* antigens (TpN47, TpN17, and TpN15) and one synthetic peptide (TmpA). Serum or plasma samples were diluted at a ratio of 1:100 and incubated with antigen-coated strips at room temperature overnight, followed by incubation with an alkaline phosphatase-labeled anti-human IgG conjugate. After washing, a chromogen substrate was added for visualization of reactive bands. Band intensities were scored semi-qualitatively (0–4) relative to control lines, and results were interpreted as positive, negative, or indeterminate based on the number and intensity of reactive bands. Data interpretation was performed using LiRAS for Infectious Diseases v4.00 CE.

#### 2.6.4. Rapid Plasma Reagin (RPR) Test

The RPR card test (Fortress Diagnostics Ltd., Antrim, Northern Ireland, UK) is a qualitative and semi-quantitative non-treponemal flocculation assay used to detect reagin antibodies. The serum sample is mixed with the antigen on a card, and the presence of visible agglutination is interpreted as a reactive result. Reactive samples are further tested by serial dilution to determine antibody titers for semi-quantitative analysis. The assay was conducted according to the manufacturer’s instructions [38].

### 2.7. Statistical Analysis

Statistical analyses were performed using GraphPad Prism (version 9, San Diego, CA, USA). Agreement-based performance measures, including sensitivity, specificity, positive predictive value (PPV), negative predictive value (NPV), overall percent agreement (OPA), positive percent agreement (PPA), and negative percent agreement (NPA), were calculated. Sensitivity and specificity were estimated relative to classification within the routine diagnostic algorithm using established comparator assays, rather than against a single definitive reference standard. Accordingly, performance metrics reflect concordance and discordance between assays within this framework. Given the use of preselected and archived samples, these estimates are intended to reflect comparative assay agreement within the study design rather than population-level diagnostic performance. Ninety-five percent confidence intervals (95% CI) were calculated for all performance measures. Samples yielding indeterminate results on the comparator assay were excluded from the primary performance analysis and considered separately. Concordance between assays was assessed using Cohen’s kappa coefficient. Differences in paired discordant proportions between assays were evaluated using McNemar’s test. A *p*-value < 0.05 was considered statistically significant [39,40,41,42,43,44,45].

## 3. Results

### 3.1. Performance of the MAGLUMI^®^ HIV Ab/Ag Combi Assay in Comparison with INNO-LIA^®^ HIV I/II Score

A comparative analysis was conducted to evaluate the performance of the MAGLUMI^®^ HIV Ab/Ag Combi assay relative to the antibody differentiation assay INNO-LIA^®^ HIV I/II Score within the study sample set. Among the 240 HIV specimens analyzed, 38 (15.8%) were classified as positive and 173 (72.1%) as negative by INNO-LIA^®^ HIV I/II Score, while 28 samples (11.7%) yielded indeterminate results (Table 1). Table 1. Comparison of MAGLUMI HIV and INNO-LIA HIV results within the diagnostic algorithm (*n* = 240).

Among samples with definitive (positive and negative) INNO-LIA^®^ HIV I/II Score results, all 38 INNO-LIA-positive samples were reactive by MAGLUMI^®^ HIV Ab/Ag Combi. Of the 174 INNO-LIA-negative samples, 173 were non-reactive by MAGLUMI HIV, while one yielded an indeterminate MAGLUMI result. No discordant reactive MAGLUMI^®^ HIV results were observed among INNO-LIA^®^ HIV negative samples. Among the 28 INNO-LIA^®^ HIV indeterminate samples, MAGLUMI^®^ HIV classified 26 as non-reactive, one as reactive, and one as indeterminate (Table 1).

After excluding indeterminate results, 211 samples were included in the concordance analysis. The MAGLUMI^®^ HIV Ab/Ag Combi assay demonstrated a sensitivity of 100.0% (95% CI: 90.7–100%), specificity of 100.0% (95% CI: 97.9–100%), and overall percent agreement (OPA) of 100.0% (95% CI: 98.3–100%). Cohen’s kappa coefficient indicated complete agreement (κ = 1.00) between MAGLUMI^®^ HIV Ab/Ag Combi and INNO-LIA^®^ HIV I/II Score (Table 2). These findings reflect concordance with the antibody differentiation assay within the diagnostic algorithm and should be interpreted in the context of comparator-based classification rather than as a measure of absolute diagnostic accuracy.

### 3.2. Performance of the MAGLUMI^®^ HIV Ab/Ag Combi Assay Within the PCR-Tested Subset

In accordance with the diagnostic algorithm used in the Medical Commission screening program, HIV-1 RNA PCR testing was performed only for specimens reactive by the ARCHITECT^®^ HIV screening assay. Within this subset of ARCHITECT-reactive samples (*n =* 80), PCR detected HIV-1 RNA in 36 (45.0%) samples, while 44 (55.0%) were PCR negative (Table S1). Among the PCR-negative samples, four were positive by INNO-LIA^®^ HIV I/II Score, representing discordant serological and molecular findings within this subset. The complete multi-modality results for all 44 ARCHITECT-reactive samples with discordant findings across ARCHITECT^®^, INNO-LIA^®^, MAGLUMI^®^, and PCR are presented in Table S2, with samples grouped according to INNO-LIA^®^ HIV I/II Score result and CDC 2014 algorithm classification. The performance of the MAGLUMI^®^ HIV assay was evaluated relative to PCR within this ARCHITECT-reactive subset. Among 79 samples with definitive MAGLUMI results, 35 PCR-positive samples were reactive by MAGLUMI^®^ HIV, and 40 PCR-negative samples were non-reactive (Table 3). These findings corresponded to a positive concordance rate of 100% (95% CI: 90.0–100%), a negative concordance rate of 90.9% (95% CI: 78.3–97.5%) with HIV-1 RNA PCR, and an overall percent agreement (OPA) of 94.9%. Cohen’s kappa coefficient indicated almost high agreement between MAGLUMI^®^ HIV and HIV-1 RNA PCR (κ = 0.90). These estimates reflect agreement within a preselected subset of ARCHITECT-reactive samples. Because PCR testing was restricted to ARCHITECT-reactive samples, these estimates may not reflect overall screening performance in unselected populations.

### 3.3. Performance of the VITROS^®^ ECiQ HIV Combo Assay in Comparison with INNO-LIA^®^ HIV I/II Score

The performance of the VITROS^®^ *ECiQ* HIV Combo assay was evaluated relative to INNO-LIA^®^ HIV I/II Score within the study sample set. Among the 179 samples tested, 37 (20.7%) were classified as positive and 115 (64.2%) as negative by INNO-LIA^®^ HIV I/II Score, while the remaining samples yielded indeterminate results. Among samples with definitive (positive or negative) INNO-LIA^®^ HIV results, all INNO-LIA^®^ HIV positive samples were reactive by VITROS^®^ HIV, with no discordant non-reactive results observed in this group. However, five samples were reactive by VITROS^®^ HIV but negative by INNO-LIA^®^ HIV I/II Score, representing discordant reactive results within this comparison (Table 4). These findings reflect agreement within the comparator-based diagnostic framework rather than an independent determination of infection status.

After exclusion of indeterminate and insufficient-volume samples, 157 specimens were included in the concordance analysis. The VITROS^®^HIV Combo assay showed an estimated sensitivity of 100% (95% CI: 90.5–100%), specificity of 95.8% (95% CI: 90.5–98.6%), and overall percent agreement (OPA) of 96.8% (95% CI: 92.7–99.0). Cohen’s kappa coefficient indicated excellent agreement with INNO-LIA^®^ HIV I/II Score (κ = 0.916) (Table 5). These estimates reflect agreement within this subset and within the comparator-based diagnostic framework rather than an independent determination of infection status.

### 3.4. Performance of the VITROS^®^ ECiQ HIV Combo Assay Within the PCR-Tested Subset

The VITROS^®^ ECiQ HIV Combo assay was evaluated relative to PCR among ARCHITECT-reactive samples with available results. Of the 76 specimens tested by VITROS^®^ HIV, 35 were PCR-positive and 41 were PCR-negative. Among the PCR- positive samples, all 35 were reactive by VITROS^®^ HIV Combo. Among the PCR-negative samples, 30 were non-reactive and 11 were reactive, representing discordant reactive results within this subset. These findings corresponded to a positive concordance rate of 100% and a negative concordance rate of 73.2% with HIV-1 RNA PCR, with an overall percent agreement of 85.5%. Cohen’s kappa coefficient indicated substantial agreement between VITROS^®^ ECiQ HIV Combo and HIV-1 RNA PCR (κ = 0.715; 95% CI: 0.556–0.864) (Table 6). These estimates reflect agreement within this subset and within the comparator-based diagnostic framework rather than an independent determination of infection status. Because PCR testing was restricted to ARCHITECT-reactive samples, these estimates may not reflect overall screening performance in unselected populations.

### 3.5. Characterization of Discordant Serological and Molecular Patterns in the ARCHITECT-Reactive PCR-Evaluated Subset

In interpreting the discordant patterns described in this section, the CDC 2014 HIV Diagnostic Algorithm was applied as the classification framework [13]. Within this algorithm, a positive result on the HIV-1/HIV-2 antibody differentiation assay (INNO-LIA^®^ HIV I/II Score) at Step 2 constitutes confirmed HIV infection, and HIV-1 RNA PCR is reserved exclusively for resolving cases with indeterminate or negative antibody differentiation results; it is not a required step following a confirmed positive antibody differentiation result. Accordingly, all classifications in this section reflect CDC algorithm-based interpretation.

To further characterize the discordant patterns observed within the ARCHITECT-reactive PCR-evaluated subset, the results of all five testing modalities, ARCHITECT^®^ HIV Ag/Ab Combo, MAGLUMI^®^ HIV Ab/Ag Combi, VITROS^®^ ECiQ HIV Combo, INNO-LIA^®^ HIV I/II Score, and HIV-1 RNA PCR, were examined in parallel for the 11 samples yielding discordant results across Groups A, B1, and B2 (Table S3). An additional two samples (PP402 and PP438) identified as Group C are described separately below.

Four distinct patterns were identified (Figure 2). In Group A (*n* = 4; PP421, PP422, PP423, PP426), all three immunoassays were reactive, INNO-LIA^®^ was positive, and PCR was negative. In Group B1 (*n* = 3; PI406, PI412, PI415), ARCHITECT^®^ and VITROS^®^ were reactive while MAGLUMI^®^ was non-reactive, INNO-LIA^®^ yielded persistently indeterminate results at both initial testing and repeat testing at four weeks, and PCR was negative at both timepoints. In Group B2 (*n* = 4; PN406, PN407, PN414, PN419), ARCHITECT^®^ and VITROS^®^ were reactive while MAGLUMI^®^ was non-reactive, INNO-LIA^®^ was negative, and PCR was negative. In Group C (*n* = 2; PP402, PP438), INNO-LIA^®^ HIV I/II Score yielded indeterminate results while HIV-1 RNA PCR was positive, consistent with acute HIV infection during the early seroconversion stage.

In Group A, the positive INNO-LIA^®^ result confirms HIV infection at Step 2 of the CDC 2014 algorithm in all four cases, irrespective of the negative PCR result. Per the algorithm, PCR is not required following a confirmed positive antibody differentiation result and its negativity does not alter the HIV-positive classification [13]. These findings are consistent with controlled viremia, including ART-suppressed viremia, elite controller status, or sub-threshold viremia below the PCR detection limit (20 copies/mL), as discussed in Section 4.2 [13,46,47]. Importantly, all three immunoassay platforms, ARCHITECT^®^, MAGLUMI^®^, and VITROS^®^, successfully detected all four confirmed HIV-positive cases despite the negative PCR result, demonstrating the clinical value of fourth-generation Ag/Ab immunoassays in identifying confirmed HIV-seropositive individuals with controlled viremia.

In Group B2, the negative INNO-LIA^®^ result at Step 2 and the negative PCR result at Step 3 together indicate no laboratory evidence of HIV infection per the complete CDC 2014 algorithm [13]. The reactive results produced by ARCHITECT^®^ and VITROS^®^ in all four Group B2 cases therefore represent false-reactive signals within the diagnostic algorithm, as they were not supported by either antibody differentiation or molecular testing. MAGLUMI^®^ was non-reactive in all four cases, concordant with the algorithm-based classification. In Group B1, the persistently indeterminate antibody differentiation results at both timepoints with continued PCR negativity precluded definitive classification within the study period; per the CDC 2014 algorithm, extended clinical follow-up is required for these cases [13]. MAGLUMI^®^ was non-reactive in all three Group B1 cases, consistent with the absence of confirmed seroconversion.

Across all seven Group B cases, both ARCHITECT^®^ and VITROS^®^ were reactive in all seven, while MAGLUMI^®^ was non-reactive in all seven. These findings demonstrate that MAGLUMI^®^ HIV Ab/Ag Combi produced fewer false-reactive results than both ARCHITECT^®^ HIV Ag/Ab Combo and VITROS^®^ ECiQ HIV Combo within this discordant subset, showing superior concordance with the final CDC algorithm-based classification.

A fourth pattern was identified in Group C (*n* = 2). In these two cases, INNO-LIA^®^ HIV I/II Score yielded indeterminate results while HIV-1 RNA PCR was positive, confirming active viral replication consistent with acute HIV infection during the early seroconversion stage. Per the CDC 2014 algorithm, a positive PCR result in the context of an indeterminate antibody differentiation result confirms HIV-1 infection [13]. ARCHITECT^®^ HIV Ag/Ab Combo and VITROS^®^ ECiQ HIV Combo were reactive in both Group C cases (2/2). MAGLUMI^®^ HIV Ab/Ag Combi was reactive in one case and yielded a result of 3 AU/mL in the other, which the instrument software classified as indeterminate. As 3 AU/mL exceeds the MAGLUMI^®^ non-reactive threshold of 1 AU/mL, this result represents a detectable signal above the assay cutoff. Per the manufacturer’s interpretation algorithm (Snibe Diagnostics Co. Ltd., personal communication, 2026), values between 1 and 4 AU/mL are classified as weak reactive, with persistent borderline results reported as indeterminate by the instrument software (Supplementary Figure S1). Accordingly, all three immunoassay platforms produced signals above the non-reactive threshold in both Group C cases, demonstrating their ability to detect p24 antigen during the early seroconversion stage. Notably, no cases of INNO-LIA^®^ negative with PCR-detected HIV-1 RNA were identified among the 80 ARCHITECT^®^-reactive samples (Figure 2), indicating that all confirmed HIV infections in this cohort had developed at least a partial serological response detectable by antibody differentiation testing at the time of screening.

### 3.6. Performance of the ARCHITECT^®^ Syphilis TP Assay in Comparison with INNO-LIA^®^-Syphilis Score

The performance of the automated treponemal assay ARCHITECT^®^ Syphilis TP was evaluated relative to INNO-LIA^®^ Syphilis Score within the study sample set. Among the 180 samples analyzed, 39 (21.7%) were classified as positive, and 139 (77.2%) as negative by INNO-LIA^®^ Syphilis Score, while the remaining samples yielded indeterminate results (Table 7). Among samples with definitive (positive or negative) INNO-LIA^®^ Syphilis Score results, all INNO-LIA-positive and INNO-LIA-negative samples showed concordant results with ARCHITECT^®^ Syphilis TP, with no discordant reactive and non-reactive results observed in this set. These findings reflect agreement within this subset and within the comparator-based diagnostic framework rather than an independent determination of infection status.

### 3.7. Performance of the MAGLUMI^®^ Syphilis Assay in Comparison with INNO-LIA^®^ Syphilis Score

The performance of the MAGLUMI^®^ Syphilis assay was evaluated relative to the INNO-LIA^®^ Syphilis Score within the same sample set. Among the 180 samples tested, 39 (21.7%) were classified as positive and 138 (76.7%) as negative by INNO-LIA^®^ Syphilis Score, while the remaining samples yielded indeterminate results. Among samples with definitive (positive or negative) INNO-LIA^®^ Syphilis Score results, all samples showed concordant results with the MAGLUMI^®^ Syphilis assay, with no discordant results observed in these groups (Table 8). These findings reflect agreement within this subset and within the comparator-based diagnostic framework rather than an independent determination of infection status.

### 3.8. Concordance Between the MAGLUMI^®^ Syphilis and ARCHITECT^®^ Syphilis TP Assays

The concordance between the MAGLUMI^®^ Syphilis and ARCHITECT^®^ Syphilis TP assays was assessed within the study sample set. Among the 180 samples evaluated, both assays yielded identical classification results across all samples, with no discordant reactive or non-reactive results observed (Table 9). These findings reflect agreement within the comparator-based diagnostic framework rather than an independent determination of infection status.

### 3.9. Comparative Performance of Treponemal Assays

The comparative performance of the MAGLUMI^®^ Syphilis and ARCHITECT^®^-Syphilis TP assays relative to the INNO-LIA^®^ Syphilis Score is summarized in Table 10. Among samples with definitive results, both assays showed estimated sensitivity, specificity, PPV, NPV, and OPA of 100.0%, with narrow confidence intervals. Cohen’s kappa analysis indicated complete agreement between each assay and INNO-LIA^®^ Syphilis Score (*κ* = 1.00). These estimates reflect agreement within this subset and within the comparator-based diagnostic framework rather than an independent determination of infectious status.

### 3.10. Performance of the RPR Assay in Comparison with Treponemal Assays

The performance of the non-treponemal RPR assay was evaluated relative to the INNO-LIA^®^ Syphilis Score within the study sample set. Among the 180 samples analyzed, 39 (21.7%) were classified as positive and 99 (55.0%) as negative by INNO-LIA^®^ Syphilis Score, while the remaining samples yielded indeterminate or discordant results. A total of 40 samples were reactive by RPR but negative by INNO-LIA^®^ Syphilis Score (28.8%), representing discordant reactive results within this comparison. The overall percent agreement (OPA), PPV, and NPV were 77.5% (95% CI: 70.7–83.4), 49.4% (95% CI: 37.9–60.9), and 100% (95% CI: 96.3–100), respectively. The Cohen’s kappa coefficient (*κ* = 0.52) indicated moderate agreement between RPR and INNO-LIA^®^ Syphilis Score (Table 11).

Comparable patterns were observed when the RPR assay was evaluated relative to automated treponemal assays (ARCHITECT Syphilis TP and MAGLUMI^®^ Syphilis TP). Across all comparisons, the RPR assay demonstrated similar overall percent agreement (approximately 77–78%), low positive predictive values (50%), and high negative predictive values (100%), with Cohen’s kappa values indicating moderate agreement (κ = 0.52–0.53) (Table 11). These findings reinforce the observed discordant reactive pattern associated with the RPR assay across different treponemal comparators.

These findings reflect agreement within the comparator-based diagnostic framework rather than an independent determination of infection status and are consistent with the known non-specific reactivity of non-treponemal assays in this heterogeneous screening population.

## 4. Discussion

Accurate laboratory diagnosis remains essential for the control of human immunodeficiency virus (HIV) and syphilis, two infections that continue to represent significant global public health challenges. Early detection through reliable screening programs is critical for preventing transmission, initiating timely treatment, and guiding public health interventions. In this study, we evaluated the performance of automated chemiluminescent immunoassays for HIV and syphilis relative to established testing approaches within the operational context of the Medical Commission (MC) screening program in Qatar.

To our knowledge, this represents one of the first comprehensive evaluations of these platforms within a real-world, high-throughput screening setting. Overall, our findings demonstrated high levels of agreement between automated CLIA platforms and established diagnostic methods within the applied testing framework, supporting their potential utility in large-scale screening workflows.

### 4.1. Interpretation of HIV Screening Results in a Heterogeneous Screening Population

In our previous study, the ARCHITECT^®^ HIV Ag/Ab Combo assay demonstrated excellent analytical reproducibility [16]. However, its positive predictive value (PPV) was relatively low when compared with INNO-LIA^®^ HIV I/II Score and HIV-1 RNA PCR, highlighting the challenges of interpreting reactive screening results in populations with heterogeneous epidemiological backgrounds and a relatively low overall yield of confirmed infection. In the present study, only 36 of 80 ARCHITECT^®^ HIV-reactive samples (45.0%) were confirmed as HIV-1 RNA PCR positive, while 44 (55.0%) were PCR-negative (Table S1). A subset of these cases remained reactive on serological testing, reflecting discordant serological and molecular patterns. Notably, four of the PCR-negative samples were positive by INNO-LIA^®^ HIV I/II Score, leaving 40 cases that were not supported by molecular detection within the comparator framework (Table S2). These findings are consistent with the well-recognized observation that a proportion of screening-reactive results in low-prevalence settings may not be supported by supplemental testing. Such discordant serological and molecular patterns have been reported in specific clinical scenarios, including individuals receiving antiretroviral therapy (ART) with suppressed viral load and elite controllers, in whom HIV RNA may remain below the limit of detection despite persistent antibody response [46,47]. Similar observations have been reported in studies evaluating fourth-generation HIV immunoassays [16,48,49]. Collectively, these results reaffirm that ARCHITECT^®^ HIV Ag/Ab Combo, while operationally robust, may generate a meaningful proportion of non-confirmed reactive results in large-scale settings, with implications for cost, workload, and reporting timelines.

### 4.2. Comparative Performance of Automated HIV CLIA Platforms

The MAGLUMI^®^ HIV Ab/Ag Combi assay showed excellent concordance with INNO-LIA^®^ HIV I/II Score, with complete agreement among samples with definitive results (κ = 1.00) (Table 1 and Table 2). Within the PCR-evaluated subset, MAGLUMI^®^ HIV Ab/Ag Combi assay maintained a positive concordance rate of 100% with PCR and demonstrated a higher negative concordance rate than the VITROS^®^ ECiQ HIV Combo, with fewer discordant reactive results observed within this enriched subset. The MAGLUMI^®^ HIV assay also resolved the majority of INNO-LIA^®^ HIV indeterminate results. This reduction in indeterminate outcomes has important operational implications, as indeterminate serologic profiles frequently require repeat testing and patient recall, thereby increasing laboratory workload and prolonging reporting times. Together, these findings suggest that MAGLUMI^®^ HIV assay may reduce the burden of discordant and indeterminate results encountered with ARCHITECT^®^ HIV-based screening workflows, with potential implications for laboratory efficiency and downstream confirmatory testing requirements.

The VITROS^®^ ECiQ HIV Combo assay similarly demonstrated strong agreement with INNO-LIA^®^ HIV I/II Score; however, within the PCR-evaluated subset, it demonstrated a lower negative concordance rate with HIV-1 RNA PCR than MAGLUMI^®^ HIV Ab/Ag Combi, reflecting a higher proportion of discordant reactive results within this enriched ARCHITECT-reactive subset. This lower negative concordance rate warrants careful interpretation in the context of the assay’s analytical design. The VITROS^®^ ECiQ HIV Combo employs a two-stage sandwich immunometric technique in which HIV antigens and antibodies are captured on streptavidin-coated wells, a format that is inherently susceptible to interference from heterophilic antibodies, which can bridge between reagent antibodies and produce false-reactive signals in sandwich immunoassays [50]. The manufacturer’s package insert explicitly acknowledges that samples with total protein greater than 9 g/dL and those containing heterophilic antibodies may yield falsely reactive results [34]. In the heterogeneous MC screening population, comprising expatriate workers from diverse geographic and clinical backgrounds, the prevalence of such interfering factors may be higher than in homogeneous clinical populations in which the assay was originally validated. Additionally, the streptavidin-coated well format uses biotinylated capture reagents that are susceptible to interference from elevated free biotin levels in patient samples [51]. However, it is noted that ARCHITECT^®^ HIV Ag/Ab Combo, which does not employ biotin–streptavidin capture, demonstrated an identical false-reactive pattern to VITROS^®^ in the Group B discordant cases characterized in Section 3.5, suggesting that heterophilic antibody susceptibility shared by both sandwich-format immunoassays represents a more consistent explanatory mechanism than biotin interference alone [17,50]. Importantly, PCR and Ag/Ab combination assays detect fundamentally different biological targets. Several well-characterized clinical scenarios, including ART-suppressed viremia, elite controller status, and sub-threshold viremia below the assay’s limit of detection (20 copies/mL), may produce Ag/Ab-reactive, PCR-negative patterns representing confirmed HIV-seropositive individuals with controlled viremia rather than platform-level false reactivity [13,47,52]. The observed discordance arose within a pre-selected ARCHITECT-reactive subset, which should be considered when interpreting these findings. In such cases, the inclusion of antibody differentiation testing as an intermediate step, as mandated by the CDC 2014 diagnostic algorithm, is essential for accurate classification, since it is this step that distinguishes confirmed seropositive individuals with controlled viremia from genuine false-reactive immunoassay signals [13]. In contrast, as demonstrated in Section 3.5, MAGLUMI^®^ HIV Ab/Ag Combi was non-reactive in all seven cases where HIV infection was unconfirmed or excluded by the CDC 2014 algorithm, consistent with its lower susceptibility to non-specific binding in this challenging subset. Taken together, and as further evidenced by the five-modality parallel analysis described in Section 3.5, these analytical and population-level factors may collectively explain the discordant patterns observed with VITROS^®^ in the PCR-evaluated subset, though definitive attribution is precluded by the absence of clinical metadata in this retrospective study.

The five-modality parallel analysis of the 11 discordant samples yields two clinically important findings regarding immunoassay platform performance and the necessity of algorithm-based HIV interpretation.

First, in Group A (*n* = 4), all three immunoassay platforms successfully detected all four cases confirmed HIV-positive by antibody differentiation testing at Step 2 of the CDC 2014 algorithm [13], despite negative HIV-1 RNA PCR. Per the algorithm, PCR plays no role in classifying cases with a positive antibody differentiation result; its negativity in these four cases reflects controlled viremia rather than the absence of infection, as discussed above [13,47,52]. Had PCR been used as the sole classification criterion, all four confirmed HIV-seropositive individuals would have been missed. This finding is of direct clinical and public health significance: it demonstrates unequivocally that all three fourth-generation Ag/Ab immunoassay platforms can detect confirmed HIV-seropositive individuals with suppressed or sub-threshold viremia in a diverse screening population. It further reinforces that PCR negativity alone cannot exclude HIV infection in the context of positive antibody differentiation [13,14].

Second, in Group B (*n* = 7), MAGLUMI^®^ HIV Ab/Ag Combi produced fewer false-reactive results than both ARCHITECT^®^ HIV Ag/Ab Combo and VITROS^®^ ECiQ HIV Combo. Both ARCHITECT^®^ and VITROS^®^ were reactive in all seven Group B cases where HIV infection was either unresolved (Group B1, *n* = 3) or excluded by the algorithm (Group B2, *n* = 4), while MAGLUMI^®^ was non-reactive in all seven. The differential false-reactive performance between the platforms is likely attributable to platform-specific differences in antigen coating architecture, signal threshold calibration, and susceptibility to non-specific binding. Both ARCHITECT^®^ and VITROS^®^ are sandwich-format immunoassays susceptible to heterophilic antibody interference, a mechanism that operates independently of the specific capture technology and has been documented specifically for ARCHITECT^®^ HIV Ag/Ab Combo [17,50]. VITROS^®^ additionally employs a biotin–streptavidin capture design susceptible to biotin-related interference [34,51]. However, since ARCHITECT^®^, which does not use biotin, showed an identical false-reactive pattern across all seven Group B cases, heterophilic antibody susceptibility is the more consistent shared mechanism explaining the false-reactive behavior of both platforms. MAGLUMI^®^’s lower false-reactive rate in this subset likely reflects differences in its magnetic microbead antigen coating density and signal threshold calibration, resulting in more stringent discrimination between specific and non-specific binding in this challenging cohort. Importantly, none of the three platforms missed any confirmed HIV-positive case in Group A, indicating that the observed performance difference is specific to false-reactive discrimination rather than sensitivity.

Together, these findings support two conclusions: first, that all three fourth-generation Ag/Ab immunoassay platforms successfully identified confirmed HIV-positive cases with controlled viremia that PCR missed, demonstrating their clinical indispensability in algorithm-based screening; and second, that MAGLUMI^®^ demonstrated superior discriminatory performance by producing fewer false-reactive results in this discordant subset. Furthermore, these findings reinforce that antibody differentiation testing is the indispensable step within the CDC diagnostic algorithm; without it, the four confirmed HIV-positive cases in Group A would have been classified alongside the seven false-reactive Group B cases as a single undifferentiated group of PCR-negative immunoassay-reactive specimens, making accurate clinical classification impossible [13,14].

Group C (*n* = 2) represents a clinically important and distinct pattern, INNO-LIA^®^ indeterminate with positive PCR, consistent with acute HIV infection during the early seroconversion stage, in which p24 antigen and viral RNA are detectable but the antibody response has not yet matured sufficiently for a definitive positive antibody differentiation result [13,47,52]. Critically, all three fourth-generation immunoassay platforms produced signals above the non-reactive threshold in both Group C cases. ARCHITECT^®^ HIV Ag/Ab Combo and VITROS^®^ ECiQ HIV Combo were reactive in both cases, while MAGLUMI^®^ HIV Ab/Ag Combi yielded a reactive result in one case and an indeterminate result (3 AU/mL) in the other. Per the manufacturer’s interpretation algorithm (Snibe Diagnostics Co. Ltd., personal communication, 2026), values between 1 and 4 AU/mL represent weak reactive signals, reported as indeterminate when persistent (Supplementary Figure S1), confirming that the 3 AU/mL result represents a detected signal rather than a missed result. This finding demonstrates that all three fourth-generation Ag/Ab combination assays retained sensitivity in the acute window period, a capability that third-generation antibody-only assays lack entirely. Furthermore, these two cases underscore the indispensable role of PCR in the CDC 2014 algorithm, without PCR, the indeterminate INNO-LIA^®^ result would not have permitted definitive classification, and both acute infections would have remained unresolved.

The identification of Group C cases carries important implications for screening program design. In high-throughput public health screening settings such as the Medical Commission program, the use of fourth-generation Ag/Ab combination immunoassays, which simultaneously detect both HIV-1 p24 antigen and HIV-1/2 antibodies, provides a critical advantage over third-generation antibody-only assays by narrowing the diagnostic window period. Third-generation assays rely exclusively on antibody detection and would not produce a reactive signal during the early seroconversion stage, when circulating p24 antigen is present but the antibody response has not yet developed. In contrast, all three fourth-generation platforms evaluated in this study produced signals above the non-reactive threshold in both Group C cases, confirming their ability to flag acute HIV infection for further molecular investigation. Without a fourth-generation screening assay, both Group C cases would have been reported as negative, the reflex PCR testing would never have been triggered, and two individuals with active HIV infection would have been missed entirely at the point of screening. These findings provide direct empirical evidence, from a real-world high-throughput public health screening program, that fourth-generation Ag/Ab immunoassays are not merely analytically superior to third-generation assays but are clinically indispensable in settings where early detection of acute HIV infection is a public health priority.

Importantly, both assays were assessed under identical conditions; the observed performance differences therefore reflect relative platform behavior within this challenging enriched subset. Findings should not be directly extrapolated to unselected screening populations. Despite this limitation, the assay’s automation, standardization, and suitability of these assays for high-throughput workflows remain advantageous for large clinical laboratories, where operational capacity and rapid turnaround are critical. Overall, both MAGLUMI^®^ HIV Ab/Ag Combi and VITROS^®^ ECiQ HIV Combo represent viable alternatives to ARCHITECT^®^ HIV Ag/Ab Combo platform for HIV screening within this context; however, within the evaluated Medical Commission context, MAGLUMI^®^ HIV Ab/Ag Combi demonstrated more consistent performance in reducing discordant and INNO-LIA–associated indeterminate results.

### 4.3. Performance of Treponemal Syphilis Assays

Syphilis serology remains the cornerstone of laboratory diagnosis. Non-treponemal assays such as RPR are clinically valuable for reflecting disease activity and monitoring treatment response. However, these assays detect anti-cardiolipin antibodies rather than pathogen-specific antibodies and are therefore susceptible to biological non-specific reactivity. In contrast, treponemal assays detect antibodies directed against *Treponema pallidum*-specific antigens and generally remain reactive for life following infection, limiting their ability to distinguish active from past infection. Many laboratories have adopted reverse sequence testing algorithms, beginning with automated treponemal immunoassays followed by non-treponemal testing for reactive samples. This approach improves diagnostic efficiency and supports detection of latent infection [14]. Current guidance recommends additional treponemal testing for discordant patterns such as immunoassay reactive with non-reactive RPR results [11,49]. In screening settings where disease prevalence is low, selecting assays with high specificity is particularly important to minimize discordant reactive results and their downstream consequences.

The widely used Abbott ARCHITECT^®^ Syphilis TP assay, known for its automation capabilities, high throughput, and strong analytical performance [53,54,55,56,57], demonstrated excellent agreement with the INNO-LIA^®^ Syphilis Score in our study, with estimated sensitivity and specificity of 100% among samples with definitive results. These findings are consistent with previous studies reporting sensitivity and specificity values exceeding 98% [58]. Additionally, Tao et al. reported a PPV of 88.25% and an NPV of 99.97% [58]. In our study, higher PPV and NPV estimates were observed, which may reflect differences in study population characteristics, sample selection, and diagnostic algorithm.

The MAGLUMI^®^ Syphilis immunoassay demonstrated comparable agreement with the INNO-LIA^®^ Syphilis Score, with estimated specificity and sensitivity of 100% among samples with definitive results. No discordant reactive or non-reactive results were observed in this subset. In this study, the MAGLUMI^®^ Syphilis assay demonstrated operational suitability for high-volume screening settings, with a testing duration of approximately 18 min, supporting its potential integration into high-throughput diagnostic workflows.

Among the evaluated samples, a small number of discordant or indeterminate patterns were observed across the treponemal assays. One sample yielded an indeterminate INNO-LIA^®^ Syphilis Score result with reactivity to the TpN17 antigen, while both MAGLUMI^®^ Syphilis and ARCHITECT^®^ Syphilis TP were reactive, which may reflect early or borderline treponemal reactivity. This is consistent with the reported high sensitivity of TpN17 antigen in early infection [27,59]. Another sample was non-reactive by A RCHITECT^®^ Syphilis TP and negative by INNO-LIA^®^ Syphilis Score but showed a borderline reactivity on MAGLUMI^®^ Syphilis, which may represent non-specific reactivity. Notably, this sample also demonstrated a reactive result in the RPR test. A third sample demonstrated non-reactive results across all assays except for an indeterminate INNO-LIA^®^ Syphilis Score result associated with the TmpA antigen, suggesting possible antigen-related cross-reactivity. Previous studies have shown that certain antigens, including TmpA, may cross-react with sera from patients with various clinical conditions, such as mononucleosis, hepatitis, diabetes mellitus, HIV/AIDS, and advanced age, which may explain the discordant findings observed in the second and third cases [60,61,62].

### 4.4. Performance and Limitations of the RPR Assay

The Medical Commission currently employs a traditional screening algorithm, using the RPR test as the initial screening step, followed by confirmation with a treponemal assay such as ARCHITECT^®^ Syphilis TP [59,63]. Our previous study raised concerns regarding the reliability of RPR as a primary screening tool due to its high rate of discordant reactive results [16]. Consistent with those findings, the present study demonstrated that RPR exhibited a PPV of 49.4% and moderate agreement with INNO-LIA^®^ Syphilis Score (κ = 0.52). Approximately 29% of RPR-reactive samples were not supported by treponemal testing within the comparator-based framework, reinforcing the limitations of using RPR as a primary screening tool in this context and highlighting the challenges of interpreting reactive screening results in heterogeneous screening populations with diverse epidemiological backgrounds (Table 11). The high false-positive rate and reduced PPV of the RPR assay observed in the present study reflect well-established limitations of non-treponemal tests in heterogeneous screening populations. Unlike treponemal assays, the RPR detects anticardiolipin antibodies produced in response to lipoidal antigens released from damaged host cells, a mechanism that is not specific to *Treponema pallidum* infection. Consequently, biological false-positive (BFP) reactions are well-documented across a broad range of non-syphilitic conditions [64,65,66]. These include autoimmune disorders (particularly systemic lupus erythematosus and antiphospholipid syndrome), acute bacterial and viral infections, malaria, tuberculosis, pregnancy, and polyclonal B-cell activation. The MC screening population comprises expatriate workers from diverse geographic origins, including regions where malaria, tuberculosis, hepatitis, and other endemic infections with known capacity to trigger non-specific anticardiolipin antibody production are prevalent. This epidemiological heterogeneity is a major contributor to the high BFP rate observed. Furthermore, the very low prevalence of active syphilis in this population, estimated at approximately 0.09% among migrant workers in Qatar [67], mathematically ensures that even a modest reduction in specificity disproportionately lowers PPV, as has been previously established for non-treponemal tests across settings of varying prevalence [68]. These findings are consistent with our previous report from the same MC population, in which the PPV of RPR against treponemal confirmatory testing was 36.6% [16], and collectively support the case for transitioning to a reverse sequence syphilis screening algorithm beginning with an automated treponemal CLIA assay. These findings underscore the importance of confirmatory treponemal testing and cautious interpretation of isolated RPR reactivity.

### 4.5. Operational and Public Health Implications

Beyond analytical agreement, operational considerations are critical in large-scale screening programs. Traditional algorithms requiring repeated testing and multiple confirmatory steps increase reagent utilization, laboratory workload, and associated costs. Although a formal cost-effectiveness analysis was not performed in this study, automated chemiluminescent platforms such as MAGLUMI^®^ and VITROS^®^ ECiQ may reduce indirect operational burden by decreasing repeat testing and minimizing indeterminate outcomes. Improved assay specificity may therefore contribute to fewer confirmatory assays and more efficient utilization of laboratory resources, particularly in national screening programs where large populations are tested annually. While implementation of automated platforms may require initial capital investment, the observed reduction in discordant and indeterminate results with MAGLUMI^®^ assay suggests that these upfront costs may be offset over time through decreased confirmatory testing, reduced patient recall procedures, and streamlined laboratory workflows.

### 4.6. Limitations

This study has several limitations. First, the sample size for certain analytical subsets, particularly PCR-tested samples and VITROS^®^ evaluation, was constrained by specimen availability and volume limitations. Second, the sample set was intentionally enriched with discordant and indeterminate profiles, to support method comparison; however, this design limits the ability to estimate predictive values in an unselected screening population. Third, clinical metadata—including symptoms, disease stage, treatment history, and follow-up results—were not available, limiting definitive interpretation of discordant findings. Proviral DNA testing was also not performed, precluding molecular characterization of PCR-negative but serologically reactive cases.

Fourth, infection status was defined according to the existing diagnostic algorithm rather than through independent clinical review or a universal composite reference standard. Furthermore, this was a single-center study, and a formal cost-effectiveness analysis was not conducted. Fifth, the high biological false-positive rate observed with the RPR assay could not be attributed to specific clinical conditions, as serological data regarding autoimmune disorders, concurrent infections, pregnancy status, or geographic disease exposure were not available for individual samples. Future studies incorporating clinical metadata would allow more precise characterization of the sources of RPR biological false-positive reactivity in this population. Future prospective studies incorporating clinical follow-up, proviral DNA assessment, and direct comparison of traditional versus reverse syphilis screening algorithms in MC-style screening populations would further refine diagnostic workflows and optimize public health impact. It should also be noted that HIV-1 RNA PCR testing was performed only on ARCHITECT-reactive specimens in accordance with the diagnostic algorithm used in the Medical Commission screening program.

Finally, this study evaluated the performance of the automated immunoassay platforms specifically within the context of HIV-1 detection. HIV-2 infection was not represented in the study cohort; however, it should be noted that the Medical Commission screening population comprises expatriate workers from diverse geographic origins, including regions of sub-Saharan Africa and West Africa, where HIV-2 is endemic. Although the overall prevalence of HIV-2 remains low in this setting, its potential presence in a geographically heterogeneous screening population cannot be excluded. The performance of these platforms for HIV-2 detection therefore warrants evaluation, particularly in high-throughput screening programs serving populations from HIV-2 endemic regions.

## 5. Conclusions

This study provides a comprehensive evaluation of automated chemiluminescent immunoassays for HIV and syphilis detection within a high-throughput public health screening program.

For HIV testing, the MAGLUMI^®^ HIV Ab/Ag Combi assay demonstrated excellent agreement through the INNO-LIA^®^-HIV I/II Score and maintained high performance within the PCR-evaluated subset of ARCHITECT-reactive samples. The VITROS^®^ ECiQ HIV Combo also demonstrated high agreement with the INNO-LIA^®^ HIV I/II Score, although with a lower negative concordance rate with HIV-1 RNA PCR than MAGLUMI^®^ HIV Ab/Ag Combi in the PCR-evaluated subset.

The analysis of discordant profiles across all five testing modalities yielded three additional findings of direct clinical significance. First, all three fourth-generation Ag/Ab immunoassay platforms, ARCHITECT^®^, MAGLUMI^®^, and VITROS^®^, successfully detected all four confirmed HIV-positive cases by antibody differentiation testing at Step 2 of the CDC 2014 algorithm, despite negative HIV-1 RNA PCR, demonstrating the ability of immunoassay-based screening to identify confirmed HIV-seropositive individuals with controlled viremia below the PCR detection threshold [13]. Second, MAGLUMI^®^ HIV Ab/Ag Combi produced fewer false-reactive results than both ARCHITECT^®^ and VITROS^®^ in the discordant subset, being non-reactive in all seven cases where HIV infection was unconfirmed or excluded by the algorithm, compared with reactive results from both ARCHITECT^®^ and VITROS^®^ across the same seven cases. Third, two cases identified as Group C showed INNO-LIA^®^ indeterminate results with positive HIV-1 RNA PCR, consistent with acute HIV infection during the early seroconversion stage; all three fourth-generation immunoassay platforms produced signals above the non-reactive threshold in both cases, confirming their ability to detect p24 antigen during the pre-antibody window period. These findings collectively reinforce that no single result, including HIV-1 RNA PCR, is sufficient for definitive HIV classification, and that antibody differentiation testing remains the indispensable step within the diagnostic algorithm for accurate resolution of discordant immunoassay and molecular findings [13,14]. Together, these findings support three conclusions: first, that all three fourth-generation Ag/Ab immunoassay platforms successfully identified confirmed HIV-positive cases with controlled viremia that PCR missed, demonstrating their clinical indispensability in algorithm-based screening; second, that MAGLUMI^®^ demonstrated superior discriminatory performance by producing fewer false-reactive results in this discordant subset; and third, that all three fourth-generation platforms retained sensitivity during the acute seroconversion window period, demonstrating the clinical superiority of Ag/Ab combination assays over third-generation antibody-only platforms in high-throughput public health screening programs.

For syphilis testing, both MAGLUMI^®^ Syphilis and ARCHITECT^®^ Syphilis TP exhibited excellent agreement with the INNO-LIA^®^ Syphilis Score, whereas the RPR assay demonstrated limited positive predictive value (49.4%) and moderate (κ = 0.52) agreement, consistent with well-established biological false-positive reactivity in heterogeneous screening populations and supporting consideration of a reverse sequence syphilis screening algorithm at the Medical Commission.

Collectively, these findings suggest that automated chemiluminescent immunoassay platforms may serve as practical alternatives within high-throughput screening workflows. Differences in performance were observed across platforms, particularly with respect to discordant and indeterminate results, with the MAGLUMI^®^ assay demonstrating more consistent performance in reducing these outcomes in this study. The integration of such assays into screening algorithms may improve diagnostic efficiency and reduce unnecessary confirmatory testing, with potential benefits for workflow optimization in large-scale programs such as those operated by Qatar’s Medical Commission. These findings may also be relevant to comparable screening settings internationally.

## Figures and Tables

**Table 1:** Comparison of MAGLUMI^®^ HIV Ab/Ag Combi and INNO-LIA^®^ HIV I/II Score results within the diagnostic algorithm (*n* = 240).

MAGLUMI^®^ HIV Ab/Ag Combi	INNO-LIA^®^ HIV I/II Score Positive	INNO-LIA^®^ HIV I/II Score Negative	INNO-LIA^®^ HIV I/II Score Indeterminate	Total
Reactive	38 (15.8%)	0 (0.0%)	1 (0.4%)	39 (16.3%)
Non-reactive	0 (0.0%)	173 (72.1%)	26 (10.8%)	199 (82.9%)
^‡^ Indeterminate	0 (0.0%)	1 (0.4%)	1 (0.4%)	2 (0.8%)
Total	38 (15.8%)	174 (72.5%)	28 (11.7%)	240 (100%)

Values are presented as a number (percentage). Classification is based on comparator assay results within the diagnostic algorithm and does not represent a definitive reference standard. ^‡^ One MAGLUMI^®^ HIV Ab/Ag Combi result of 3 AU/mL was classified as indeterminate by the instrument software, consistent with the manufacturer’s algorithm for weak reactive results (1–4 AU/mL). This value exceeds the non-reactive threshold of 1 AU/mL and represents a detectable signal above the assay cutoff (Supplementary Figure S1).

**Table 2:** Agreement-based performance of the MAGLUMI^®^ HIV Ab/Ag Combi assay in comparison with INNO-LIA^®^ HIV I/II Score within the diagnostic algorithm (*n* = 211).

Test	Comparator	Sensitivity% (95% CI)	Specificity% (95% CI)	OPA% (95% CI)	PPV% (95% CI)	NPV% (95% CI)	PPA% (95% CI)	NPA% (95% CI)	Kappa (95% CI)
**MAGLUMI^®^ HIV Ab/Ag Combi**	INNO-LIA^®^ HIV I/II Score	100 (90.7–100)	100 (97.9–100)	100 (98.3–100)	100 (90.7–100)	100 (97.9–100)	100 (90.7–100)	100 (97.9–100)	1.00 (1.00–1.00)

Values are presented as a number (percentage). Classification is based on comparator assay results within the diagnostic algorithm and does not represent a definitive reference standard. A total of 28 samples with indeterminate INNO-LIA^®^ HIV I/II Score results were excluded from the primary analysis; exclusion of these samples may influence performance estimates.

**Table 3:** Agreement-based performance of the MAGLUMI^®^ HIV Ab/Ag Combi assay relative to HIV-1 RNA PCR within the ARCHITECT-reactive subset (*n* = 80).

Test	Comparator			Sensitivity% (95% CI)	Specificity% (95% CI)	OPA% (95% CI)	PPV% (95% CI)	NPV% (95% CI)	PPA% (95% CI)	NPA% (95% CI)	Kappa (95% CI)
MAGLUMI^®^ HIV Ab/Ag Combi	PCR Positive	PCR Negative	Total								
Reactive	35 (43.8%)	4 (5.0%)	39 (48.8%)	100 (90.0–100)	90.9 (78.3–97.5)	94.9 (87.5–98.6)	89.7 (75.8–97.1)	100 (91.2–100)	100 (90.0–100)	90.9 (78.3–97.5)	0.90 (0.80–1.00)
Non-Reactive	0 (0.0%)	40 (50.0%)	40 (50.0%)								
^‡^ Indeterminate	1 (1.25%)	0 (0.0%)	1 (1.25%)								
Total	36 (45.0%)	44 (55.0%)	80 (100%)								

Values are presented as a number (percentage). PCR testing was performed only on ARCHITECT-reactive samples in accordance with the diagnostic algorithm. Samples with indeterminate MAGLUMI HIV results were excluded from performance calculations. Performance measures are based on comparator classification within the diagnostic algorithm and do not represent absolute diagnostic accuracy. Percentages are calculated relative to the total number of samples (*n* = 80). ^‡^ One MAGLUMI^®^ HIV Ab/Ag Combi result of 3 AU/mL was classified as indeterminate by the instrument software, consistent with the manufacturer’s algorithm for weak reactive results (1–4 AU/mL). This value exceeds the non-reactive threshold of 1 AU/mL and represents a detectable signal above the assay cutoff (Supplementary Figure S1).

**Table 4:** Comparison of VITROS^®^ ECiQ HIV Combo and INNO-LIA^®^ HIV I/II Score results within the diagnostic algorithm (*n* = 179).

Test	Comparator			Total
VITROS^®^ ECiQ HIV Combo	INNO-LIA^®^ HIV I/II Score Positive	INNO-LIA^®^ HIV I/II Score Negative	INNO-LIA^®^ HIV I/II Score Indeterminate	
Reactive	37 (20.7%)	5 (2.8%)	5 (2.8%)	47 (26.3%)
Non-Reactive	0 (0.0%)	155 (64.2%)	17 (9.5%)	132 (73.7%)
Total	37 (20.7%)	120 (67.0%)	22 (12.3%)	179 (100%)

Values are presented as a number (percentage). Classification is based on comparator assay results within the diagnostic algorithm and does not represent a definitive reference standard. Percentages are calculated relative to the total sample size (*n* = 179).

**Table 5:** Agreement-based performance of the VITROS^®^ ECiQ HIV Combo assay in comparison with INNO-LIA^®^ HIV I/II Score within the diagnostic algorithm (*n* = 157).

Test	Comparator	Sensitivity% (95% CI)	Specificity% (95% CI)	OPA% (95% CI)	PPV% (95% CI)	NPV% (95% CI)	PPA% (95% CI)	NPA% (95% CI)	Kappa (95% CI)
**VITROS^®^ ECiQ HIV Combo**	INNO-LIA^®^ HIV I/II Score	100 (90.5–100)	95.8 (90.5–98.6)	96.8 (92.7–99.0)	88.1 (74.4–96.0)	100 (96.8–100)	100 (90.5–100)	95.8 (90.5–98.6)	0.92 (0.84–0.99)

Values are presented as a number (percentage). Classification is based on comparator assay results within the diagnostic algorithm and does not represent a definitive reference standard. A total of 22 samples with indeterminate results by INNO-LIA^®^ HIV I/II Score were excluded from the primary analysis; exclusion of these samples may influence performance estimates.

**Table 6:** Agreement-based performance of the VITROS^®^ ECiQ HIV Combo assay relative to HIV-1 RNA PCR within ARCHITECT-reactive subset (*n* = 76).

Test	Comparator			Sensitivity% (95% CI)	Specificity% (95% CI)	OPA% (95% CI)	PPV% (95% CI)	NPV% (95% CI)	PPA% (95% CI)	NPA% (95% CI)	Kappa (95% CI)
VITROS^®^ ECiQ HIV Combo	HIV-1 RNA PCR Positive	HIV-1 RNA PCR Negative	Total								
Reactive	35 (46.1%)	11 (14.5%)	46 (60.5%)	100 (90.1–100)	73.2 (57.1–85.8)	85.5 (75.6–92.5)	76.1 (61.2–87.4)	100 (88.4–100)	100 (90.0–100)	73.2 (57.1–85.8)	0.72 (0.56–0.86)
Non-reactive	0 (0.0%)	30 (39.5%)	30 (39.5%)								
Total	35 (46.1%)	41 (54.0%)	76 (100%)								

Values are presented as number (percentage). PCR testing was performed only on ARCHITECT-reactive samples in accordance with the diagnostic algorithm. Performance measures reflect agreement within this subset and do not represent absolute diagnostic accuracy. Percentages are calculated relative to the total sample size (*n* = 76).

**Table 7:** Comparison of ARCHITECT^®^ Syphilis TP and INNO-LIA^®^ Syphilis Score results within the diagnostic algorithm (*n* = 180).

Test	Comparator			Total
ARCHITECT^®^ Syphilis TP	INNO-LIA^®^ Syphilis Score Positive	INNO-LIA^®^ Syphilis Score Negative	INNO-LIA^®^ Syphilis Score Indeterminate	
Reactive	39 (21.7%)	0 (0.0%)	1 (0.6%)	40 (22.2%)
Non-Reactive	0 (0.0%)	139 (77.2%)	1 (0.6%)	140 (77.8%)
Total	39 (21.7%)	139 (77.2%)	2 (1.1%)	180 (100%)

Values are presented as a number (percentage). Classification is based on comparator assay results within the diagnostic algorithm and does not represent a definitive reference standard. Percentages are calculated relative to the total sample size (*n* = 180).

**Table 8:** Comparison of MAGLUMI^®^ Syphilis and INNO-LIA^®^ Syphilis Score results within the diagnostic algorithm (*n* = 180).

Test	Comparator			Total
MAGLUMI^®^ Syphilis	INNO-LIA^®^ Syphilis Score Positive	INNO-LIA^®^ Syphilis Score Negative	INNO-LIA^®^ Syphilis Score Indeterminate	
Reactive	39 (21.7%)	0 (0.0%)	1 (0.6%)	40 (22.2%)
Non-Reactive	0 (0.0%)	138 (76.7%)	1 (0.6%)	139 (77.2%)
Indeterminate	0 (0.0%)	1 (0.6%)	0 (0.0%)	1 (0.6%)
Total	39 (21.7%)	139 (77.2%)	2 (1.1%)	180 (100%)

Values are presented as a number (percentage). Classification is based on comparator assay results within the diagnostic algorithm and does not represent a definitive reference standard.

**Table 9:** Comparison of MAGLUMI^®^ Syphilis and ARCHITECT^®^ Syphilis TP results within the diagnostic algorithm (*n* = 180).

Test	Comparator		Total
MAGLUMI^®^ Syphilis	ARCHITECT^®^ Syphilis TP Reactive	ARCHITECT^®^ Syphilis TP Non-Reactive	
Reactive	40 (22.2%)	0 (0.0%)	40 (22.2%)
Non-Reactive	0 (0.0%)	139 (77.2%)	139 (77.2%)
indeterminate	0 (0.0%)	1 (0.6%)	1 (0.6%)
Total	40 (22.2%)	140 (77.8%)	180 (100%)

Values are presented as a number (percentage). Classification is based on comparator assay results within the diagnostic algorithm and does not represent a definitive reference standard.

**Table 10:** Agreement-based comparative performance of treponemal assays within the diagnostic algorithm (*n* = 178).

Test	Comparator	Sensitivity% (95% CI)	Specificity% (95% CI)	PPV% (95% CI)	NPV% (95% CI)	PPA% (95% CI)	NPA% (95% CI)	OPA% (95% CI)	Cohen’s Kappa Coefficient (95% CI)
ARCHITECT^®^ Syphilis TP	INNO-LIA^®^ Syphilis Score	100 (91–100)	100 (97.4–100)	100 (91–100)	100 (97.4–100)	100 (91–100)	100 (97.4–100)	100 (97.9–100)	1.00
MAGLUMI^®^ Syphilis	INNO-LIA^®^ Syphilis Score	100 (91–100)	100 (97.4–100)	100 (91–100)	100 (97.4–100)	100 (91–100)	100 (97.4–100)	100 (97.9–100)	1.00
MAGLUMI^®^ Syphilis	ARCHITECT^®^ Syphilis TP	100 (91.2–100)	100 (97.4–100)	100 (91.2–100)	100 (97.4–100)	100 (91.2–100)	100 (97.4–100)	100 (98–100)	1.00

Values are presented as numbers (percentage), where applicable. Classification is based on comparator assay results within the diagnostic algorithm and does not represent a definitive reference standard. Samples with indeterminate results were excluded from the primary analysis; exclusion of these samples may influence performance estimates.

**Table 11:** Agreement-based comparison of RPR with treponemal assays within the diagnostic algorithm (*n* = 180).

**RPR vs. ARCHITECT^®^ Syphilis TP**								
**RPR Results**	**Comparator Reactive**	**Comparator Non-reactive**	**PPV%**	**NPV%**	**PPA%**	**NPA%**	**OPA%**	**Cohen’s Kappa Coefficient (κ)**
Reactive	40 (22.2%)	40 (22.2%)	50 (38.6–61.4)	100 (96.4–100)	100 (91.2–100)	71.4 (63.2–78.7)	77.8 (71–83.6)	0.53 (0.41–0.64)
Non-Reactive	0 (0.00%)	100 (55.6%)						
**RPR vs. INNO-LIA^®^ Syphilis Score**								
**RPR results**	**Comparator Positive**	**Comparator Negative**	**PPV%**	**NPV%**	**PPA%**	**NPA%**	**OPA%**	**Cohen’s Kappa Coefficient (κ)**
Reactive	39 (21.9%)	40 (22.5%)	49.4 (37.9–60.9)	100 (96.3–100)	100 (91–100)	71.2 (62.9–78.6)	77.5 (70.7–83.4)	0.52 (0.41 to 0.64)
Non-Reactive	0 (0%)	99 (55.6%)						
**RPR vs. MAGLUMI^®^ Syphilis**								
**RPR results**	**Comparator Reactive**	**Comparator Non-reactive**	**PPV%**	**NPV%**	**PPA%**	**NPA%**	**OPA%**	**Cohen’s Kappa Coefficient (κ)**
Reactive	40 (22.2%)	39 (21.8%)	50.6 (39.1–62.1)	100 (96.4–100)	100 (91.2–100)	71.9 (63.7–79.2)	78.2 (71.4–84)	0.53 (0.42–0.65)
Non-Reactive	0 (0.00%)	100 (55.9%)						

Values are presented as number (percentage), where applicable. Classification is based on comparator assay results within the diagnostic algorithm and does not represent a definitive reference standard. Performance measures reflect agreement within the study sample set.

## Data Availability

The original contributions presented in this study are included in the article/Supplementary Material. Further inquiries can be directed to the corresponding authors.

## Supplementary Materials

The following supporting information can be downloaded at: https://www.mdpi.com/article/10.3390/microorganisms14061302/s1, Figure S1: MAGLUMI^®^ HIV Ab/Ag Combi result interpretation algorithm for borderline reactive results. Source: Snibe Diagnostics Co. Ltd., provided to the authors upon request (personal communication, 2026); Table S1: Concordance of ARCHITECT^®^ HIV Ag/Ab Combo with HIV-1 RNA PCR within the ARCHITECT-reactive subset (*n* = 80); Table S2: Multi-modality results for 44 ARCHITECT^®^ HIV Ag/Ab Combo-reactive samples with discordant findings classified by INNO-LIA^®^ HIV I/II Score (*n* = 44); Table S3: Parallel five-modality results for 11 discordant samples in the ARCHITECT-reactive PCR-evaluated subset with CDC 2014 algorithm classification (*n* = 11).
