# Supplementary material for: Strengthening STD Screening Programs: Comprehensive Evaluation of High-Throughput Immunoassays for HIV and Syphilis Detection
Source: Microorganisms. 2026 Jun 9;14(6):1302. doi: 10.3390/microorganisms14061302 (PMC13304136; doi:10.3390/microorganisms14061302)

**Table S1.** Comparative results of ARCHITECT® HIV Ag/Ab Combo and HIV-1 RNA PCR within the ARCHITECT-reactive subset (n=80)

|  | PCR-HIV + | PCR-HIV - | Total | PPV | PPA |
| --- | --- | --- | --- | --- | --- |
| ARCHITECT® HIV Ag/Ab Combo Reactive | 36 (45%) | 44 (55%) | 80 (100%) | 45.0% (33.8%  -56.5%) | 100% (90.3%-100%) |
| †ARCHITECT® HIV Ag/Ab Combo Non-Reactive | NA | NA | NA |  |  |

† HIV-1 RNA PCR was performed only on ARCHITECT® HIV Ag/Ab Combo-reactive specimens in accordance with the MC diagnostic algorithm; performance metrics for non-reactive specimens are therefore not applicable.

NA: Not available

**Table S2 multi-modality results for 44 ARCHITECT® HIV Ag/Ab Combo-reactive samples with negative HIV-1 RNA PCR, classified by INNO-LIA® HIV I/II Score result (n=44)**

| **Sample No.** | **ARCHITECT® HIV Ag/Ab Combo** | **INNO-LIA® HIV I/II Score** | **MAGLUMI® HIV Ab/Ag Combi** | **HIV-1 RNA PCR** | **VITROS® ECiQ HIV Combo** |
| --- | --- | --- | --- | --- | --- |
| PP421 | Reactive | Positive | Reactive | Negative | Reactive |
| PP422 | Reactive | Positive | Reactive | Negative | Reactive |
| PP423 | Reactive | Positive | Reactive | Negative | Reactive |
| PP426 | Reactive | Positive | Reactive | Negative | Reactive |
| PI401 | Reactive | Indeterminate | Non-Reactive | Negative | Non-Reactive |
| PI402 | Reactive | Indeterminate | Non-Reactive | Negative | Non-Reactive |
| PI403 | Reactive | Indeterminate | Non-Reactive | Negative | Non-Reactive |
| PI404 | Reactive | Indeterminate | Non-Reactive | Negative | Non-Reactive |
| PI405 | Reactive | Indeterminate | Non-Reactive | Negative | Non-Reactive |
| PI406 | Reactive | Indeterminate | Non-Reactive | Negative | Reactive |
| PI407 | Reactive | Indeterminate | Non-Reactive | Negative | Non-Reactive |
| PI408 | Reactive | Indeterminate | Non-Reactive | Negative | Non-Reactive |
| PI409 | Reactive | Indeterminate | Non-Reactive | Negative | Non-Reactive |
| PI410 | Reactive | Indeterminate | Non-Reactive | Negative | Reactive |
| PI411 | Reactive | Indeterminate | Non-Reactive | Negative | Non-Reactive |
| PI412 | Reactive | Indeterminate | Non-Reactive | Negative | Non-Reactive |
| PI413 | Reactive | Indeterminate | Non-Reactive | Negative | Reactive |
| PI414 | Reactive | Indeterminate | Non-Reactive | Negative | Non-Reactive |
| PI415 | Reactive | Indeterminate | Non-Reactive | Negative | Non-Reactive |
| PI416 | Reactive | Indeterminate | Non-Reactive | Negative | Non-Reactive |
| PI417 | Reactive | Indeterminate | Non-Reactive | Negative | Non-Reactive |
| PI418 | Reactive | Indeterminate | Non-Reactive | Negative | Non-Reactive |
| PI419 | Reactive | Indeterminate | Non-Reactive | Negative | Non-Reactive |
| PI420 | Reactive | Indeterminate | Non-Reactive | Negative | Non-Reactive |
| PN401 | Reactive | Negative | Non-Reactive | Negative | Non-Reactive |
| PN402 | Reactive | Negative | Non-Reactive | Negative | Non-Reactive |
| PN403 | Reactive | Negative | Non-Reactive | Negative | Non-Reactive |
| PN404 | Reactive | Negative | Non-Reactive | Negative | Non-Reactive |
| PN405 | Reactive | Negative | Non-Reactive | Negative | Non-Reactive |
| PN406 | Reactive | Negative | Non-Reactive | Negative | Reactive |
| PN407 | Reactive | Negative | Non-Reactive | Negative | Reactive |
| PN408 | Reactive | Negative | Non-Reactive | Negative | Non-Reactive |
| PN409 | Reactive | Negative | Non-Reactive | Negative | Non-Reactive |
| PN410 | Reactive | Negative | Non-Reactive | Negative | Non-Reactive |
| PN411 | Reactive | Negative | Non-Reactive | Negative | Non-Reactive |
| PN412 | Reactive | Negative | Non-Reactive | Negative | Non-Reactive |
| PN413 | Reactive | Negative | Non-Reactive | Negative | Non-Reactive |
| PN414 | Reactive | Negative | Non-Reactive | Negative | Reactive |
| PN415 | Reactive | Negative | Non-Reactive | Negative | Non-Reactive |
| PN416 | Reactive | Negative | Non-Reactive | Negative | Non-Reactive |
| PN417 | Reactive | Negative | Non-Reactive | Negative | Non-Reactive |
| PN418 | Reactive | Negative | Non-Reactive | Negative | Non-Reactive |
| PN419 | Reactive | Negative | Non-Reactive | Negative | Reactive |
| PN420 | Reactive | Negative | Non-Reactive | Negative | Non-Reactive |

*

Classification based on CDC 2014 HIV Diagnostic Algorithm (14). MAGLUMI® HIV Ab/Ag Combi was non-reactive in all 40 Group B cases (rows 5–44) and reactive in all 4 Group A cases (rows 1–4), while ARCHITECT® HIV Ag/Ab Combo was reactive in all 44 cases. Of the 44 samples in this table, 11 discordant samples (Group A n=4, Group B1 n=3, Group B2 n=4) underwent parallel five-modality analysis and are presented separately in Table S3 (see Section 3.5).

**Table S3** ^‡^**Parallel five-modality results for 11 discordant ARCHITECT®-reactive samples with negative HIV-1 RNA PCR, with CDC 2014 algorithm classification (n=11)**

| **Sample No.** | **ARCHITECT® HIV Ag/Ab Combo** | **INNO-LIA® HIV I/II Score** | **MAGLUMI® HIV Ab/Ag Combi** | **HIV-1 RNA PCR** | **VITROS® ECiQ HIV Combo** | **Groups** |
| --- | --- | --- | --- | --- | --- | --- |
| PP421 | Reactive | Positive | Reactive | Negative | Reactive | Group A |
| PP422 | Reactive | Positive | Reactive | Negative | Reactive |  |
| PP423 | Reactive | Positive | Reactive | Negative | Reactive |  |
| PP426 | Reactive | Positive | Reactive | Negative | Reactive |  |
| PI406 | Reactive | Indeterminate | Non-Reactive | Negative | Reactive | Group B1 |
| PI412 | Reactive | Indeterminate | Non-Reactive | Negative | Reactive |  |
| PI415 | Reactive | Indeterminate | Non-Reactive | Negative | Reactive |  |
| PN406 | Reactive | Negative | Non-Reactive | Negative | Reactive | Group B2 |
| PN407 | Reactive | Negative | Non-Reactive | Negative | Reactive |  |
| PN414 | Reactive | Negative | Non-Reactive | Negative | Reactive |  |
| PN419 | Reactive | Negative | Non-Reactive | Negative | Reactive |  |

*^‡^ Classification based on CDC 2014 HIV Diagnostic Algorithm (14). Group A: INNO-LIA® positive at Step 2 = confirmed HIV infection irrespective of PCR result (controlled viremia). Group B1: INNO-LIA® indeterminate + PCR negative = unresolved (extended clinical follow-up required). Group B2: INNO-LIA® negative + PCR negative = HIV (infection excluded). Reactive = result above manufacturer cutoff; Non-Reactive = result below manufacturer cutoff*

**Supplementary Figure S1.** MAGLUMI® HIV Ab/Ag Combi result interpretation algorithm for borderline reactive results. *Source: Snibe Diagnostics Co. Ltd., provided to the authors upon request (personal communication, 2026).*


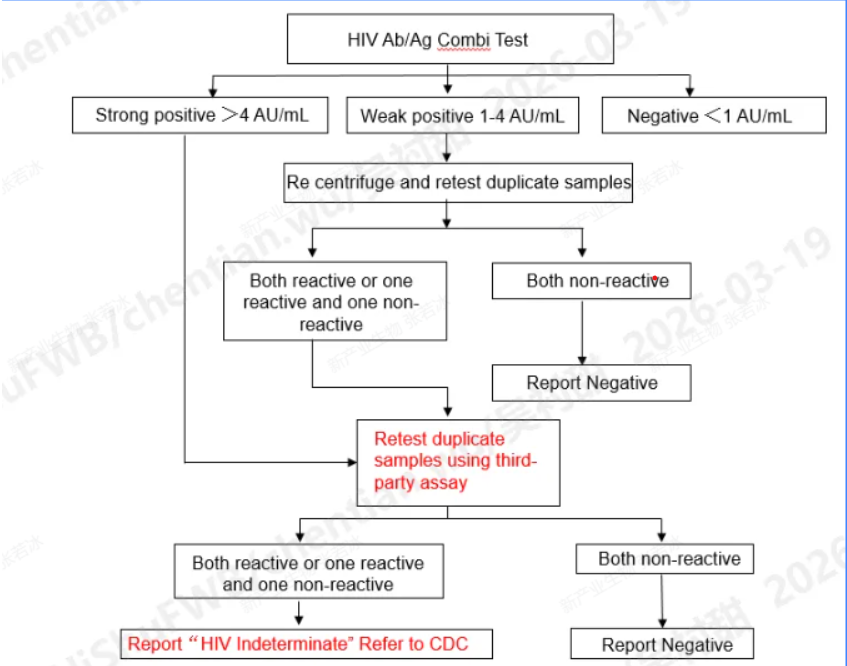

Supplement: Supplementary file 1 [file microorganisms-14-01302-s001.zip › microorganisms-4249264-supplementary.docx]
